# Supplementary figures and images for: Insights on aquatic microbiome of the Indian Sundarbans mangrove areas
Source: PLoS One. 2020 Feb 25;15(2):e0221543. doi: 10.1371/journal.pone.0221543 (PMC7041844; doi:10.1371/journal.pone.0221543)

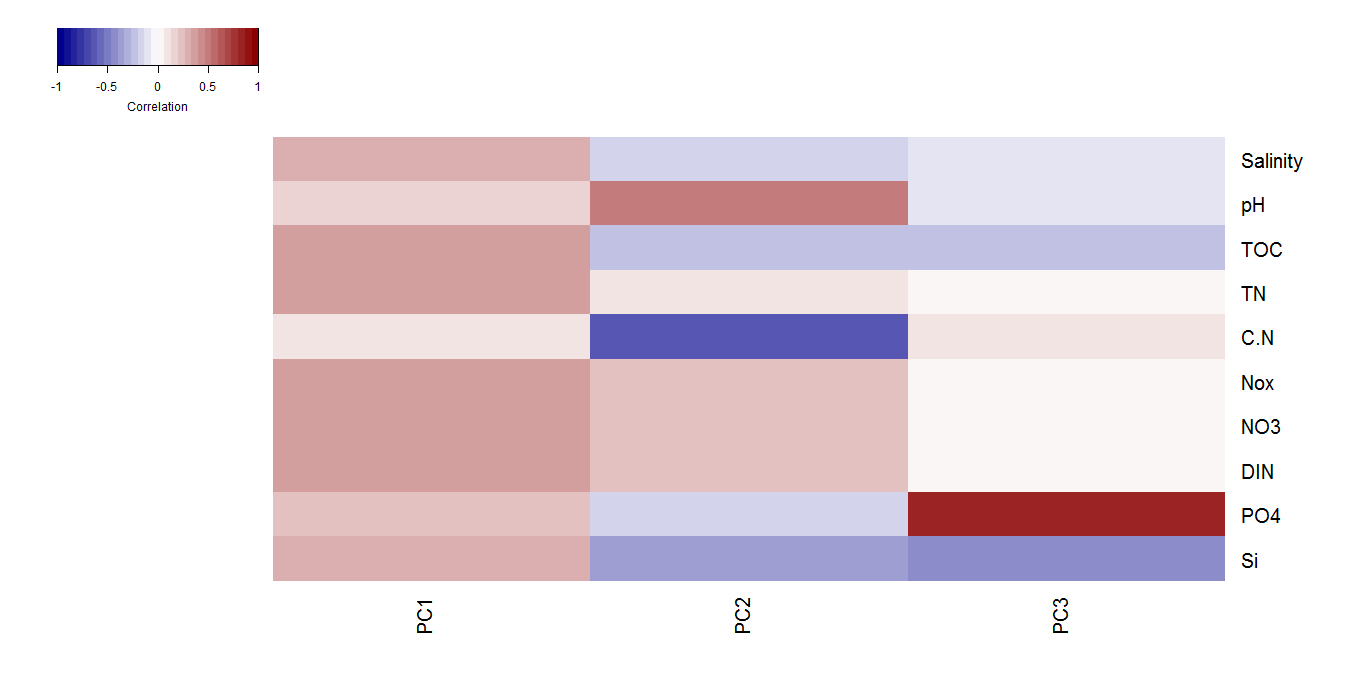

Supplement: S1 Fig — Levels of correlations are indicated with different color bar. (TIFF) [file pone.0221543.s001.tiff]

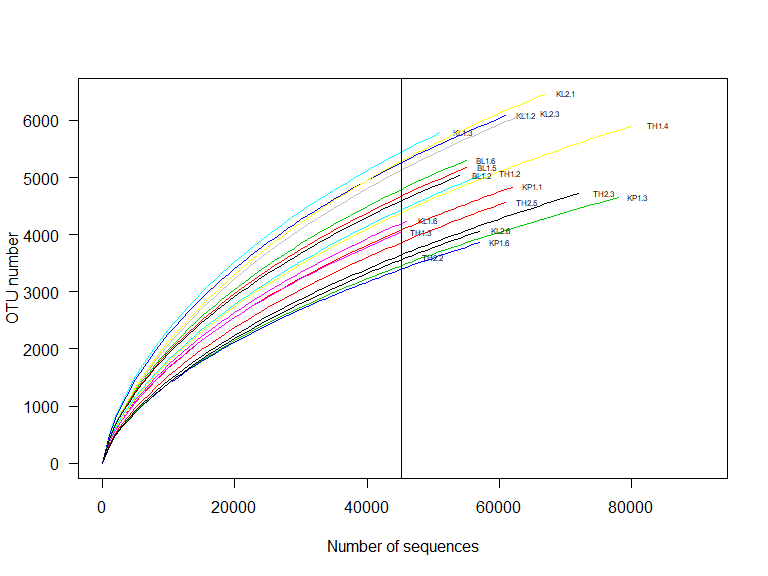

Supplement: S2 Fig — The curve approaching plateau indicates that the number of reads are enough to describe the OTUs representing the community. (TIFF) [file pone.0221543.s002.tiff]

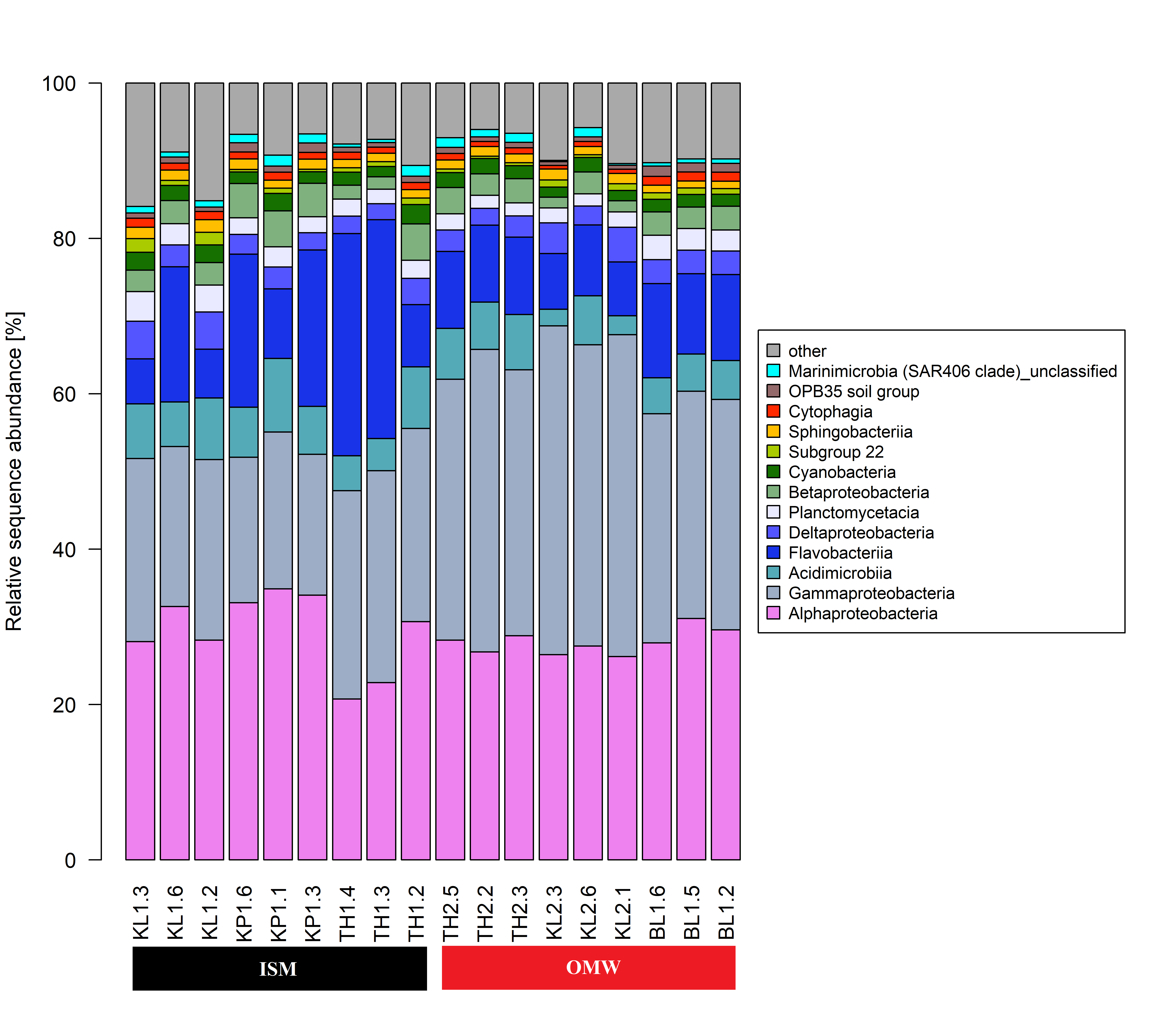

Supplement: S3 Fig — (TIF) [file pone.0221543.s003.tif]

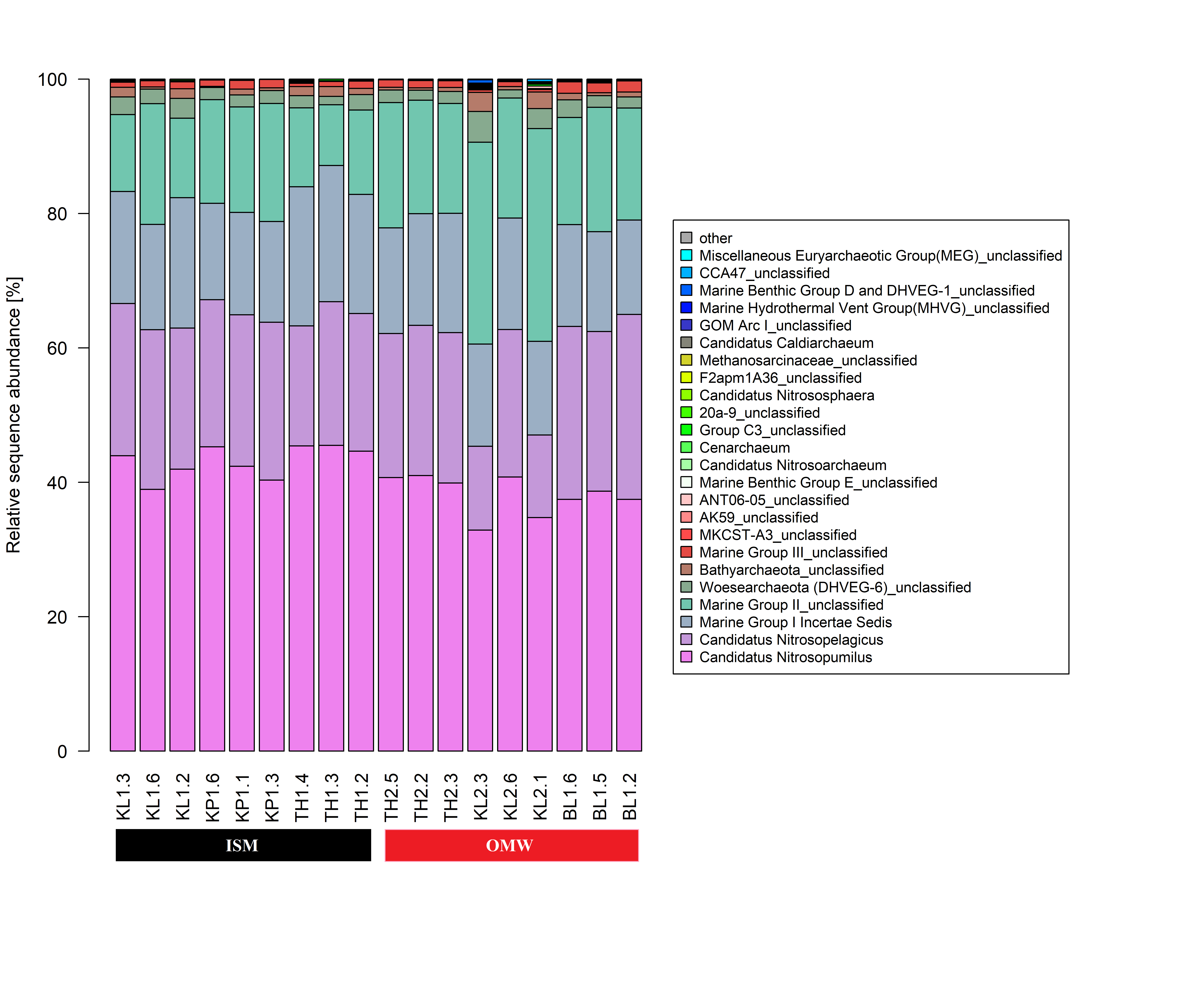

Supplement: S4 Fig — (TIF) [file pone.0221543.s004.tif]
