# Supplementary material for: Insights on aquatic microbiome of the Indian Sundarbans mangrove areas
Source: PLoS One. 2020 Feb 25;15(2):e0221543. doi: 10.1371/journal.pone.0221543 (PMC7041844; doi:10.1371/journal.pone.0221543)
Supplement: S1 Table — (DOCX) [file pone.0221543.s005.docx]

Table S1: Differences in water quality between ISM and OMW assess by GLMMs

| Parameter | Sampling stations | |
| --- | --- | --- |
|  | F | p-Value |
| pH | 1.3947 | 0.30 |
| Salinity | 7.0872 | 0.06 |
| DOC | 6.066 | 0.06 |
| TN | 10.597 | 0.03* |
| DIN | 12.288 | 0.02* |
| PO4 | 7.2748 | 0.01* |
| Si | 2.8259 | 0.16 |

(*) significant differences
