# Supplementary material for: Insights on aquatic microbiome of the Indian Sundarbans mangrove areas
Source: PLoS One. 2020 Feb 25;15(2):e0221543. doi: 10.1371/journal.pone.0221543 (PMC7041844; doi:10.1371/journal.pone.0221543)
Supplement: S2 Table — (DOCX) [file pone.0221543.s006.docx]

Table S2: Kruskal-Wallis test for alpha diversity of the two sampling station.

|  | χ2 | df | p-value | p-value adjusted wilcoxon tests |
| --- | --- | --- | --- | --- |
| Species richness | 0.095 | 1 | 0.75 | 0.79 |
| The exponential of Shannon entropy | 0.70 | 1 | 0.40 | 0.43 |
| The inverse of  Simpson index | 1.42 | 1 | 0.23 | 0.26 |
