# Supplementary material for: Insights on aquatic microbiome of the Indian Sundarbans mangrove areas
Source: PLoS One. 2020 Feb 25;15(2):e0221543. doi: 10.1371/journal.pone.0221543 (PMC7041844; doi:10.1371/journal.pone.0221543)
Supplement: Attachment — Submitted filename: Response to Reviewers.docx [file pone.0221543.s008.docx]

**Review Comments to the Author**

Please use the space provided to explain your answers to the questions above. You may also include additional comments for the author, including concerns about dual publication, research ethics, or publication ethics. (Please upload your review as an attachment if it exceeds 20,000 characters)

**Reviewer #1:**

**P1**. Lines 121-133: It said physicochemical parameters (salinity and pH), DOC, nutrient, nitrate, nitrite, combined nitrite and nitrate etc. However, they did not report measurements of TN and DIN (Table 1).

**Answer:** Yes. Its mistake. As I mentioned earlier, this work has been carried out in ZMT, Germany under expert supervision. The total nitrogen (TN) content of each the samples were measured using the EuroVector EA 3000 elemental analyzer. The DIN measurements were done based on the methods described by Grasshoff et al. 1999. (Page 6, line 127-128)

**P2**. Line 131-132: It said nitrite, nitrate, phosphate, silicate were analyzed using continuous flow analyzer. Suggest report clearly the measurements.

**Answer:** Thanks for this rising this issue and we also agree with the reviewer suggestions on including the details of this information. In this connection, this is to bring your notice that, the Dissolved inorganic nutrients that includes combined nitrate and nitrite (NOx), phosphate (PO43--P), and silicate Si(OH)4-) were analyzed using a continuous flow analyzer (Flowsys by Unity Scientific, Brookfield, USA) and detected spectrophotometrically as a colored complex (Grasshoff et al. 1999). To correct for the water color, absorption of the samples was measured without chemicals first, and the values were subtracted from those obtained with chemicals. Precision of the method was better and most of the nutrient concentrations were near the detection limit.

**P3**. Line 132: Line 132: It said ‘phosphate (PO43-)”. In Table 1, it reported TN (total nitrogen), DIN (dissolved inorganic nitrogen), and PO4. Nitrogen and phosphorus are important nutrients. Suggest check the measurement is orthophosphate (PO43-), not including pyrophosphate and metaphosphate. It is fine to use phosphate (PO43-). The orthoposphate (PO43-) is commonly used and reported as phosphate (PO43-). However, suggest check the standard solution, and check the measurement is phosphorus or phosphate. Suggest change the term here to orthophosphate-P or phosphate-P (PO43--P) to match well with DIN. Suggest check the term PO4 in Table 1. It is not correct. Suggest use orthophosphate-P, or simply use PO43--P

**Answer:** Thanks for suggestions but we are sorry for the confusion rises again. We checked with the methods again as I had little knowledge with such details. This part of comments is enriching. After getting detail information it is now clear that its actually orthophosphate (PO43-) that we measured and now in the current manuscript for both text and Table 1, this information is updated as PO_4_^3-^-P (Lines 262).

**P4.** Line 132: It said silicate (Si). In Table 1, it said Si. They are not correct. The term of Si is silicon, not silicate. Readers know silicate is important, and the measurement of silica (SiO2) is used for evaluating content of silicate. Suggest check the standard solution and measurement of this parameter. I think it is silica (SiO2), not Si, not silicate. Please check it is silica (SiO2) or silicon (Si). Suggest report silica, not silicate, not silicon.

**Answer:** Yes**.** Again thanks for raising this issue. Actually it’s a silicate (Si(OH)^4-^) that we measured. For the calibration we used Sodium hexafluorosilicate (Na_2_SiF_6_) during measurements.

**P5.** 5. Line 125 and Table 1: It said DOC and said DOC (µM). Suggest report clearly the measurement. Suggest check the unit “µM”. Suggest check the standard and report which chemical is used as standard.

**Answer:** Yes. The DOC is measured in µM. The DOC were measured by the high-temperature catalytic oxidation method on a Shimadzu TOC-VCPH total organic carbon analyser. The instrument was calibrated with a 10-point calibration curve of serial dilutions from a potassium hydrogen phthalate certified stock solution (1000 ppm Standard Fluka 76067-500ML-F). Consensus reference material provided by DA Hansell and W Chan of the University of Miami was used a positive control between every 10 samples. Each sample was measured with five replicate injections.
